# Discovery and Functional Validation of EP3 Receptor Ligands with Therapeutic Potential in Cardiovascular Disease

**DOI:** 10.3390/ijms26104879

**Published:** 2025-05-19

**Authors:** Jorge-Ricardo Alonso-Fernández, Silvia Montoro-García, Andreia-Filipa Cruz, Alicia Ponce-Valencia, Miguel Carmena-Bargueño, Horacio Pérez-Sánchez

**Affiliations:** 1 Structural Bioinformatics and High-Performance Computing Research Group (BIO-HPC), UCAM—Universidad Católica San Antonio de Murcia, Campus de Murcia, Av. de los Jerónimos, 135, 30107 Murcia, Guadalupe, Spain; 2 Health Sciences PhD Program, UCAM—Universidad Católica San Antonio de Murcia, Campus de Murcia, Av. de los Jerónimos, 135, 30107 Murcia, Guadalupe, Spain; 3 Preclinical Research of Bioactive Compounds and Drugs (PREBIOF), Izpisúa Lab, HiTech, Faculty of Health Sciences, UCAM—Universidad Católica San Antonio de Murcia, Campus de Murcia, Av. de los Jerónimos, 135, 30107 Murcia, Guadalupe, Spain; 4 Faculty of Nursing, UCAM—Universidad Católica San Antonio de Murcia, Campus de Murcia, Av. de los Jerónimos, 135, 30107 Murcia, Guadalupe, Spain

**Keywords:** screening, docking, prostaglandin, EP3, misoprostol, TUCA, masoprocol, pravastatin, hydrocortisone

## Abstract

The prostaglandin E2 receptor EP3 is emerging as a promising therapeutic target in cardiovascular diseases because of its involvement in vascular inflammation, platelet aggregation, and vasoconstriction. However, selective EP3 ligands with validated biological activities are scarce. In this study, we combined computational and experimental strategies to identify and validate novel EP3 receptor ligands with therapeutic potential. We implemented a high-throughput, structure- and ligand-based virtual screening pipeline, enabling efficient exploration of approved drugs and natural compounds from DrugBank and FooDB libraries. Top-scoring candidates were prioritised based on binding energy and pharmacophoric similarity. Selected hits were subjected to in silico ADME/Tox profiling using QikProp to identify molecules with favourable pharmacokinetic and safety parameters. TUCA, masoprocol, and pravastatin sodium have emerged as lead candidates and were validated in vitro using endothelial migration and platelet aggregation assays. TUCA exhibited the most consistent inhibitory effect on endothelial migration, whereas masoprocol and hydrocortisone significantly reduced platelet aggregation. These findings establish a multidimensional workflow for the rational identification of EP3 ligands and support their potential use in cardiovascular therapeutics.

## 1. Introduction

Cardiovascular diseases (CVD) continue to be the leading cause of death globally, with thrombotic events and endothelial dysfunction playing central roles in their pathophysiology [1]. Despite the availability of several pharmacological interventions, a substantial proportion of patients remain at a high residual risk owing to mechanisms not fully addressed by conventional therapies, such as low-grade inflammation and platelet hyperreactivity. Thus, there is an ongoing need to explore new biological pathways and pharmacological targets that could complement or improve current cardiovascular treatments [2].

Prostaglandin E2 (PGE2) and its associated receptor family (EP1–EP4) are key regulators of vascular tone, inflammation, and platelet function [3,4]. Among these, the EP3 receptor has recently attracted attention because of its unique role in promoting platelet aggregation and contributing to vasoconstriction and endothelial cell activation [5,6]. This G-protein-coupled receptor is expressed in multiple cardiovascular cell types, including endothelial cells and platelets, and mediates signalling events that favour a prothrombotic phenotype [7]. However, the EP3 receptor has historically been understudied compared to EP2 and EP4, both of which have been widely investigated for their vasodilatory and anti-inflammatory effects in vascular biology [8,9].

Recent advances in chemical biology and computational drug discovery have provided new opportunities to explore EP3-targeted ligands that are useful for obesity and insulin resistance [10,11]. Modern screening approaches, such as virtual docking and pharmacophore modelling, allow for rapid in silico evaluation of compound libraries. A previous study reported EP3 ligands, such as misoprostol, L-798,106, and 9-D1t-PhytoP [12]. Despite these technical advances, the field still lacks validated selective EP3 ligands with clear in vitro activity in disease-relevant models [13]. Moreover, the repurposing potential of existing compounds acting on EP3 has not been systematically investigated in the cardiovascular context.

The present work builds upon this gap by proposing a computational-to- experimental pipeline to identify and validate novel EP3 ligands with potential utility in vascular therapy. We hypothesised that selected natural or clinically approved molecules could functionally activate EP3, producing measurable effects on key cardiovascular processes, such as endothelial cell migration and platelet aggregation. To this end, we performed (i) blind docking and (ii) high-throughput virtual screening of structurally diverse compound libraries, integrating ligand- and structure-based approaches. A (iii) consensus from the obtained data was obtained, and promising candidates were further evaluated through ADME/Tox profiling and tested in (iv) biological assays using human endothelial cells and an ex vivo platelet model.

This study not only proposes a systematic strategy for EP3-targeted drug discovery but also provides novel insights into the potential cardiovascular roles of existing compounds, with direct implications for therapeutic repositioning. Together, these findings support the EP3 receptor as a valuable, yet underexplored target in cardiovascular pharmacology.

Our results revealed several molecules, including taurocholic acid (TUCA), masoprocol (NOGA), and pravastatin, as potential EP3 ligands with relevant anti-migratory and anti-aggregant properties.

## 2. Results

### 2.1. Blind Docking Revealed Two Distinct Potential Binding Sites on the EP3 Receptor

**Figure 1:**
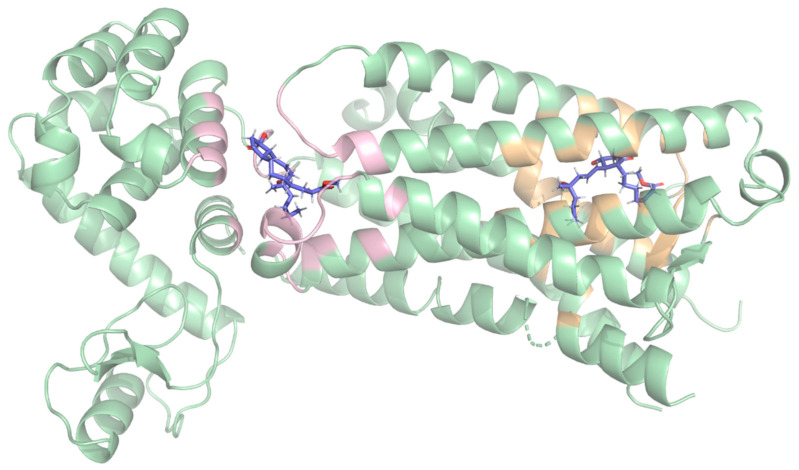
Predicted binding sites at the EP3 receptor (green) obtained from blind docking (BD), with the two top-ranked binding poses for misoprostol (blue) identified through blind docking. The pose on the right corresponds to the extracellular side (on the right, with yellow highlight), which showed the highest docking score, whereas the other pose (on the left, with pink highlight) corresponds to the cytoplasmic side.

To detect potential binding hotspots beyond the predefined ones and to compare them, unrestricted (blind) docking was performed on the receptor surface. Three ligands were selected for this technique: misoprostol, L-798,106, and 9-D1t-PhytoP. In all three cases, from the detected poses (Table S1), two high-affinity binding regions (clusters) were consistently identified, with comparable docking scores in each case (Figure 1):

The first binding region, which had slightly better docking scores, was surrounded by the following residues: P55, M58, Q103, T106, T107, V110, F133, G141, T206, W207, L329, R333, S336, and Q339. This region corresponds to the extracellular region. More details on these interactions are provided in the supplemental data (Table S2, Figure S1).

The second binding region comprised residues K83, R84, S87, A158, Y165, A166, M169, T171, I1009, K1147, R1148, and A1160. This corresponds to the cytoplasmic side.

These results suggest that the molecules may cross the membrane through this protein, which is consistent with the “transport” annotations [14]. Based on its slightly higher docking scores and relevance to extracellular ligand binding, only the first site (yellow) was selected as the target region for subsequent structure-based virtual screening analyses.

### 2.2. Prediction of Ligands by Virtual Screening

#### 2.2.1. Selection of Libraries

To obtain the first set of results via screening, two compound libraries containing known molecules were chosen: DrugBank, a database containing known drugs [15], and FooDB, a database containing compounds present in food [16]. These libraries were selected to explore both repurposing opportunities and natural-product-based leads.

#### 2.2.2. Ligand-Based Virtual Screening with LigandScout

For ligand-based screening, up to two features were allowed to be omitted during the screening process, using a pharmacophoric model of misoprostol as the query. From the DrugBank database, 10 candidate molecules emerged with one feature allowed to be omitted, including beraprost, alprostadil, isopamidol, and misoprostol. With two feature omissions, the list was expanded to 101 candidates. From the FooDB database, with one feature omission, 17 molecules emerged, including the flavonoid derivative cyanidin 3-(caffeoyl glucoside). With two feature omissions, the top-ranked hits from FooDB contained prostaglandin-like compounds.

#### 2.2.3. Structure-Based Virtual Screening with LigandScout

The structure-based virtual screening was also performed, allowing up to two omitted features. The query (pharmacophore model) (Figure S2) was based on the EP3-misoprostol interaction in the previous docking step. Only epoprostenol (a prostacyclin analogue) was matched from DrugBank with no omitted pharmacophoric features. By allowing the omission of one pharmacophoric feature, Beraprost, Alprostadil, and Dinoprost were additionally matched. When two features were allowed to be omitted, the number of potential hits increased to 19, including additional prostaglandin derivatives. From FooDB, with zero or one feature omissions, the top hits were mainly prostaglandins and derivatives. With two feature omissions, different hits emerged, such as thromboxane-related molecules.

Some visual examples for LigandScout hits are provided in the Supplementary Material (Figure S3).

#### 2.2.4. Structure-Based Virtual Screening with AutoDock Vina

The EP3 receptor structure was used for high-throughput docking with AutoDock Vina. The top-ranked compounds (with binding scores of approximately −12 kcal/mol) from DrugBank included several unnamed molecules. From FooDB, the top results included Sesamolinol, Dolineone, and a series of glycosylated isoflavones such as acetyl-/malonyl- -daidzin/-genistin.

### 2.3. Consensus, QikProp, and Compound Selection

Multiple criteria were considered to identify the most promising compounds. The table below shows the best overall compounds and their scores for all docking studies (Table 1). The criteria for their sorting and selection for experimental testing are detailed in Materials and Methods, which includes several considerations such as the existing literature on the compounds, ADME/Tox predicted properties (Table S3), commercial availability, and purchase price.

The molecules purchased for experimental testing included TUCA, hydrocortisone valerate, pravastatin sodium, and NOGA. The control molecules were iloprost, a stable prostaglandin I2 (PGI2) analogue, and PGE2. Both are FDA-approved medications and are known to affect platelets and endothelial cells.

### 2.4. Cell Viability Assay

**Figure 2:**
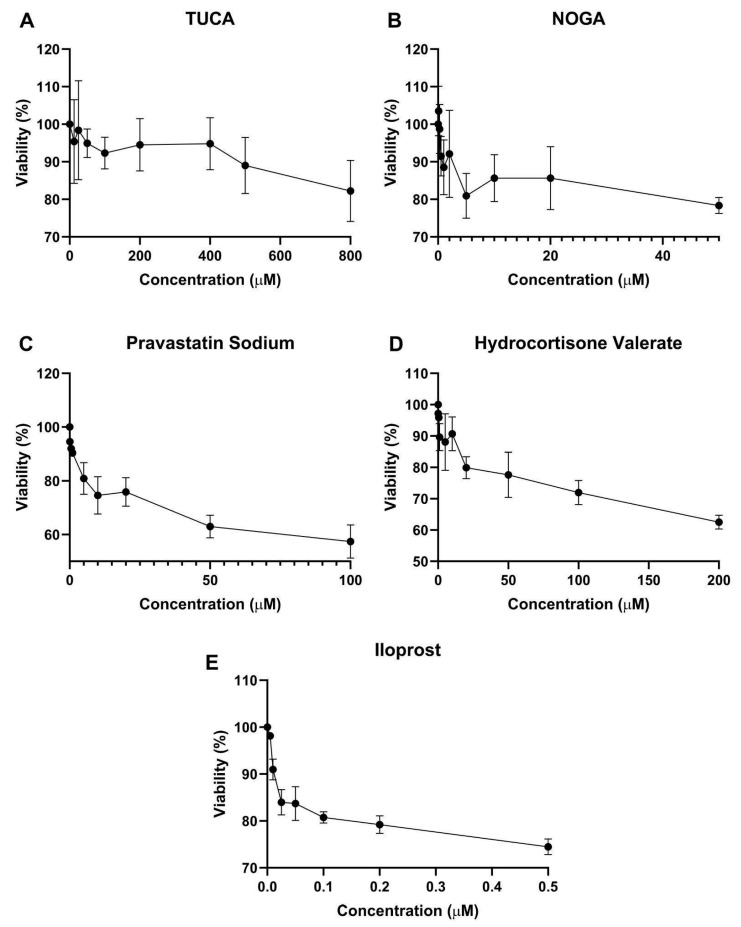
Human ES cell viability after 72 h of treatment with (**A**) TUCA, (**B**) NOGA, (**C**) Pravastatin Sodium, (**D**) Hydrocortisone Valerate, and (**E**) Iloprost. Values are mean ± SD, n = 3 independent experiments, and are expressed as the percentage of cell survival relative to the control conditions.

To determine the concentration ranges at which the compounds were not toxic to the cells, endothelial cells were treated with different concentrations, and cell viability was tested using the MTT assay after 72 h of incubation. As shown in Figure 2, maximal nontoxic concentrations (above 80% cell viability) were observed for 500 µM TUCA, 20 µM NOGA, 5 µM Pravastatin Sodium, and 20 µM hydrocortisone valerate. Iloprost significantly decreased cell viability, and then 0.2 µM was used for further in vitro studies.

### 2.5. Cell Migration Assay

The migration capacity of endothelial cells in the presence of selected EP3 ligands was determined using a wound-healing assay. This assay involves creating a standardised wound in a cell monolayer and measuring cell migration into the wound area over time. Increased migration compared with 20 nM PGE2 signifies enhanced healing and tissue regeneration. In our study, 20 nM PGE2 showed the strongest inhibition of cell migration, resulting in a 63% reduction in migration compared to the baseline migration of non-treated cells.

**Figure 3:**
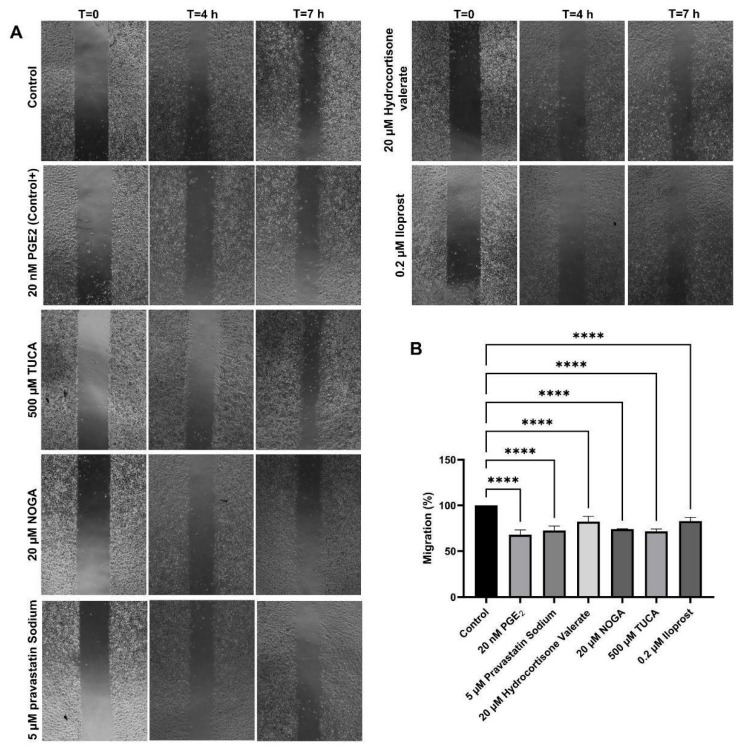
(**A**) Effect of potential EP3 ligands on endothelial cell migration after incubation for up to 7 h. (**B**) Migration values (mean ± SD, n = 3 independent experiments) are expressed as the percentage of cell migration/time relative to control conditions. An ANOVA test for parametric data was used to compare the differences between the conditions. **** *p* < 0.0001 compared with control.

The results of the scratch assay also showed that the relative migration capacity of the cells was significantly decreased in the presence of 500 µM TUCA, 20 µM NOGA, 5 µM pravastatin sodium, and 20 µM hydrocortisone valerate. The relative migration capacity was also found to be altered in the presence of 0.2 µM Iloprost (IP receptor analogue) (Figure 3).

### 2.6. Platelet Activation and Leukocyte–Platelet Aggregates Measurement Assay

Measuring platelet aggregation using flow cytometry involves assessing the extent of platelet activation before and after stimulation with ADP, together with the quantification of LPA. These approaches allowed us to evaluate the effect of preincubation with ligands on the platelet response to ADP, helping to determine how these ligands may modulate platelet activation and aggregation to leukocytes.

**Figure 4:**
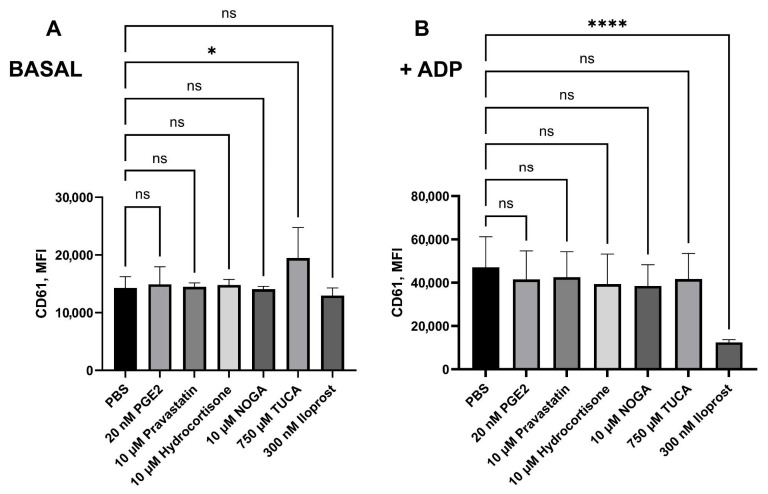
The level of platelet aggregation was expressed as CD61 expression (Mean Fluorescence Intensity, MFI), measured by flow cytometry in (**A**) basal conditions and (**B**) after 2 min of ADP (20 μM)-treated blood. Blood was pretreated with potential EP3 receptor ligands for 10 min before ADP stimulation and staining. Statistical analysis was performed using the Mann–Whitney U test. * *p* < 0.05, **** *p* < 0.0001. Data are shown as mean ± SD in the same group (n = 7). ns: nonsignificant.

We initially incubated potential EP receptor ligands for 10 min in the presence of citrated whole blood to test their effects on platelet activation. Regarding CD61’s mean fluorescence intensity (MFI), only 750 µM TUCA impaired platelet aggregation in the absence of ADP (*p* = 0.0149). Preincubation with 20 nM PGE2, 10 µM NOGA, 10 µM hydrocortisone valerate, 750 µM TUCA, or 10 µM pravastatin sodium did not significantly reverse the effect of ADP. However, treatment with 300 nM iloprost (an IP3 inhibitor) inhibited platelet aggregation (noted by a decrease in CD61 expression) in the presence of ADP (*p* < 0.0001, Figure 4).

**Figure 5:**
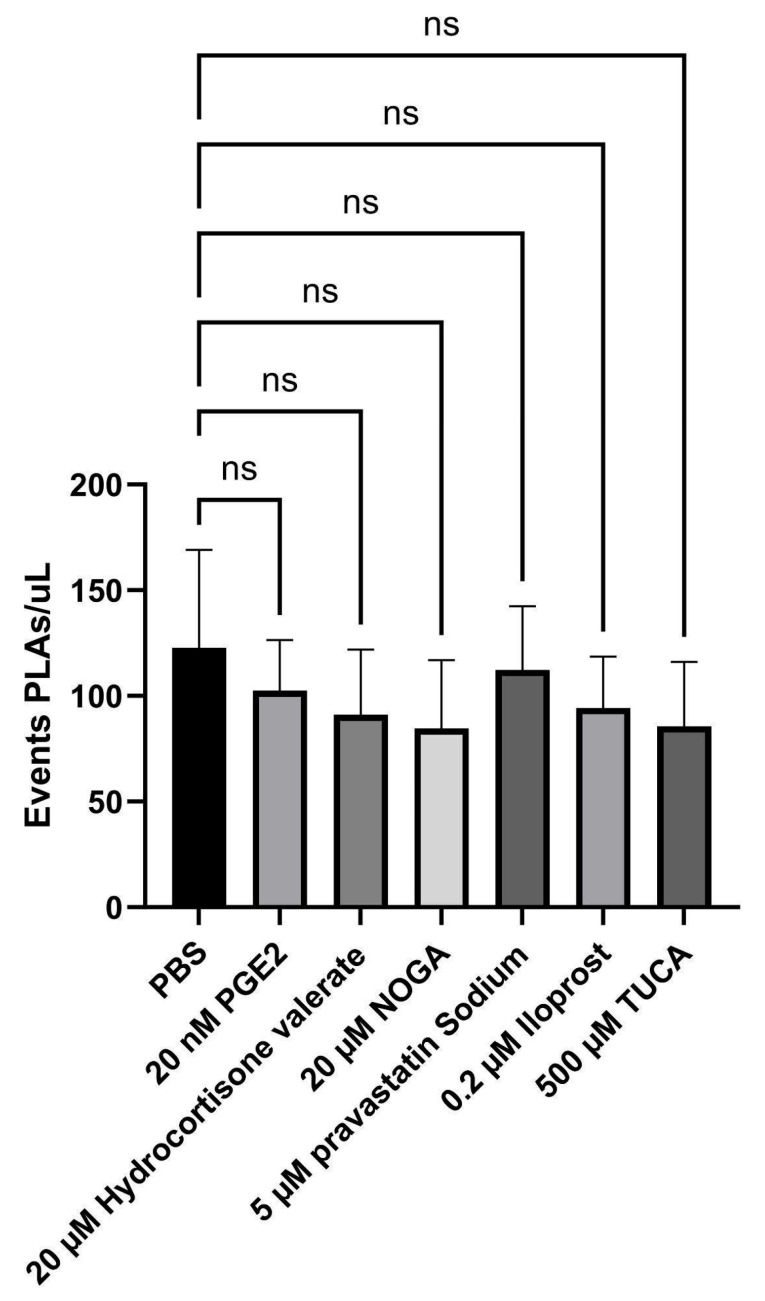
CD14+ platelet–leukocyte aggregates (PLAs) were analysed by flow cytometry in the presence of potential EP3 receptor ligands. After incubation and labelling, the blood samples were lysed, diluted, and immediately acquired on a FACS Celesta. EDTA blood samples were labelled with antibodies against CD42b-BUV395 and CD14-PE. The number of PLAs/µL is plotted as mean ± SD (n = 6). PLAs in [CD14]-gated dot plots were identified by the co-expression of CD14 and CD42b. ANOVA test for parametric data was used to compare differences between conditions (ns: nonsignificant).

Hence, to decipher the individual contribution of each EP3 receptor ligand to platelet–lymphocyte aggregates (PLAs), the events/µL were measured using flow cytometry. The addition of none of the potential EP3 receptor ligands interfered with the number of PLA events (Figure 5).

## 3. Discussion

### 3.1. Ligand Identification via Computational Methods

From the two sites identified in the blind docking, the extracellular site was already described in a previous work [12] and used here for screening, as it is the only site accessible to extracellular ligands. Although the intracellular site was not explored further in this study, future work could investigate it by performing a similar procedure of screening and experimental validation, followed by a comparative analysis with these results on the extracellular region.

In the virtual screenings performed with LigandScout, already known ligands [17] were found among the top 20 hits, asserting the validity of the pharmacophorical models and screenings: Prostaglandin D2, Alprostadil and Dinoprost were in ligand-based; Gemeprost, Cloprostenol, Misoprostol (the query model), Alprostadil (again) and Bimatoprost were in structure-based. These results reinforce the reliability of both approaches. In addition, both compounds contained several other prostaglandin derivatives.

In the screening performed with AutoDock Vina, the top compounds included several unnamed molecules; however, but among the named ones were siramesine (a sigma-2 receptor ligand [18]), sesamolinol (an antioxidant from sesame seeds [19]), and olinciguat (a soluble guanylate cyclase stimulator previously explored in cardiovascular trials [20]). These findings indicate that the docking approach not only captured structurally related prostaglandins but also retrieved chemically diverse candidates with the potential for repositioning.

Together, these in silico approaches not only yielded structurally diverse and pharmacologically plausible EP3 receptor ligands but also enabled the rational selection of candidates for biological validation. Additionally, the appearance of prostaglandins and other known ligands among the top results reinforces the validity of the computational methods applied here.

### 3.2. Biological Evaluation of Candidate Compounds

Building on computational predictions, several compounds were selected for in vitro testing to evaluate their potential biological activity in key cardiovascular processes. These assays aimed to determine the influence of EP3-targeted ligands on endothelial cell migration, platelet aggregation, and platelet–leukocyte interactions, thereby providing experimental data on their functional relevance.

The findings of this study indicate that these compounds may serve as modulators of cell migration and platelet function through their potential interactions with the EP3 receptor. Several synthetic agonists and antagonists have been developed for EP3 receptors. While agonists are used to enhance the platelet response [12,21], blocking EP3 ligands could decrease platelet or endothelial activation [22,23]. It is widely accepted that vascular prostaglandins, such as PGE_2_ and PGI_2_, play a role in the dual regulation (both enhancing and inhibiting) of cell migration through the involvement of EP and IP receptors, respectively [4,24]. PGE2 modulates angiogenesis, and 300 nM was found to promote endothelial cell migration [12], PGE2 modulates angiogenesis, and 300 nM was found to promote endothelial cell migration [12]. However, in the current study, 20 nM PGE2 hindered migration, as observed with the rest of the tested ligands. The concentration-dependent effect of PGE2 may be attributed to differential receptor binding (EP2, EP3, and EP4 are expressed in endothelial cells), leading to downstream signalling pathways [25,26]. Iloprost, a PGI_2_/prostacyclin analogue, has been shown to significantly reduce angiogenesis, although this effect was mediated through the IP receptor, as CAY10449 was able to reverse this inhibition [27]. From the current data, it was concluded that the prostacyclin mimetic iloprost could synergistically inhibit vascular cell migration, depending on a Gs-mediated increase in intracellular cAMP (IP receptor agonist and EP3 antagonist), as it could also potentially fit within the EP3 context. Iloprost has been shown to mediate vasodilatory functions via the EP4 receptor [28], and its potential binding to the EP3 receptor is an unrecognised mechanism. This illustrates that the EP3 receptor may be a novel therapeutic approach for the treatment of pulmonary arterial hypertension.

Several studies have investigated the effect of 3-hydroxy-3-methylglutaryl coenzyme A (HMG-CoA) reductase inhibition by statins on angiogenesis in vitro [29,30]. They demonstrated proangiogenic effects at low concentrations and angiostatic effects at high doses [29]. In these articles, the mechanisms behind this dual and dose-dependent effect were not attributed, which makes our study even more significant, as it is the first to link the use of pravastatin with inhibition of the EP3 receptor. In contrast, the potent antiangiogenic action of glucocorticoids, such as hydrocortisone, is mainly due to VEGF and/or prostaglandin inhibition [31]. In the present study, we found that hydrocortisone valerate could interfere with the prostanoid receptor EP3 under basal conditions. This finding is particularly interesting because no prior data regarding this interaction are available. It should be noted that pathological conditions that might influence other protective effects of these compounds (such as antioxidant or anti-inflammatory) were not considered in this study. Bile acids have been postulated to regulate endothelial metabolism by acting as protective molecules [32,33]. Taurocholic acid (TUCA), a bile acid, has been shown to reduce the endothelial migration of choroidal cells in vitro and reduce potential neovascularization [34]. Conversely, platelets inhibit thrombin activation in the presence of bile acids [35], in addition to increased fibrinolysis [36]. However, the mechanism through which this inhibition occurs is not yet understood. The fact that TUCA could be a potential ligand for the EP3 receptor may shed light on the current and previous findings.

The compounds selected for in vitro testing were clinically applied compounds, such as pravastatin, hydrocortisone, and iloprost. Our study has found an affinity for the EP3 receptor pocket, and given their clinical use, it is important to define their repositioning effects. Previous studies have found that hydrocortisone administration had no significant influence on the expression of CD62P with or without ADP stimulation [37,38]. In line with previous results, our study suggests that hydrocortisone mediates endothelial migration without activating platelet function. Elevated PLA levels are linked to various acute and chronic thromboinflammatory conditions, including CVD [39]. Therefore, they appear to be promising candidates for use as biomarkers for monitoring antiplatelet therapy. Interestingly, several bioactive compounds, such as gallic acid and anthocyanins, influence the percentage of PLA. However, only one previous study investigated a food-derived compound (phytoprostane) owing to its ability to bind to the EP3 receptor [12]. When monitoring platelet function, PLA has proven to be significantly sensitive; however, PLA levels did not change in blood from healthy populations after adding tirofiban, ADP, or TRAP [33]. Here, PGE_2_ decreased platelet reactivity in response to ADP but had no effect on the levels of PLAs, indicating the absence of platelet response to these compounds or the lack of sensitivity for PLA measurement.

Understanding the application of EP3 receptor clinical drugs, already used for other indications, can open the door to several interesting opportunities, especially in the context of drug repositioning and understanding side effects in various physiological and pathological processes. In contrast to previous findings [12], in our study, EP3 receptor potential ligands had no significant effect on platelet reactivity but decreased endothelial cell migration. In turn, the mechanism underlying such relative effects could be associated with binding to the “proangiogenic” EP3 receptor as an antagonist. Computational analysis revealed that hydrocortisone, pravastatin, and NOGA fit into the hydrophobic pocket of the EP3 receptor via hydrophobic interactions with the Met58, Thr107, Val110, and Phe133 residues as L-798,106, thus supporting its potential as an EP3 antagonist. Nonetheless, in silico analysis revealed that TUCA was slightly displaced compared to these compounds. Notably, TUCA exhibited hydrophobic and hydrogen bond interactions with residues similar to misoprostol and PGE2, including Phe140, Thr206, and Ser336. This structural alignment, coupled with its ability to stimulate platelet aggregation under basal conditions in contrast to its ability to inhibit cell migration, does not support a well-defined EP3 agonistic effect. Future approaches that offer more detailed insights into interaction dynamics, such as FRET and BRET, are thus warranted.

Our observations allow us to speculate that modulation of endothelial function might be one of the mechanisms underlying the beneficial effects of EP3 receptor ligands in a pathological context. Collectively, these findings suggest that EP3 receptor modulation can influence both the angiogenic and thrombotic pathways, highlighting its therapeutic potential in cardiovascular disease. Future studies aimed at elucidating the specific downstream signalling mechanisms of these compounds, such as the RhoA/ROCK pathway, are essential to fully understand their role. These could include experiments using animal models to evaluate their impact on thrombus formation or bleeding, angiogenesis, and other relevant cardiovascular processes. Such approaches will help to translate cellular and molecular findings into potential therapeutic applications. This potential functionality reinforces the relevance of EP3 as a pharmacological target and opens the door to repurposing clinically available agents as part of novel cardiovascular interventions. Some compounds, such as NOGA, have already been successfully reformulated to enhance their therapeutic potential, indicating that these compounds indeed have significant potential for further development and application in cardiovascular therapy [40,41].

## 4. Materials and Methods

### 4.1. Blind Docking

As the first step in the identification of novel EP3 receptor ligands, a blind molecular docking approach was employed to explore potential ligand-binding sites and assess the interaction propensities in an unbiased manner. This allows for the systematic prediction of binding poses and affinities across the receptor surface without prior assumptions about specific active sites. It also offers a rational entry point to evaluate ligand compatibility and guide the design of subsequent virtual screening and pharmacological assays.

The crystal structure of the human EP3 receptor (PDB ID: 6M9T) was prepared by removing water molecules and crystallographic ions using PyMOL [42], followed by the assignment of Gasteiger charges [43] in AutoDock Tools [44]. The receptor was then saved in the PDBQT format for docking calculations.

Three previously reported EP3 ligands [12]—misoprostol (CID 5282381), L-798,106 (CID 15551229), and 9-D1t-PhytoP (CID 126457309)—were downloaded from PubChem [45] for docking and comparison of their results. The ligand structures were processed and converted using MetaScreener to ensure compatibility with the docking pipeline. Blind docking (BD) simulations [46] were carried out with AutoDock Vina [47] through the MetaScreener suite [48]. Default settings were used to maximise reproducibility and computational efficiency. The resulting binding poses were clustered based on the distribution of docking scores, providing an estimation of binding affinities and enabling the structural grouping of potential binding modes. To further investigate the receptor–ligand interactions, the Protein–Ligand Interaction Profiler (PLIP) [49] was applied to the top-ranked complexes. This analysis facilitated the identification of key binding site residues and interaction types, which provided useful information for the preparation of pharmacophoric models during the screening step.

### 4.2. Virtual Screenings

Virtual screening (VS) techniques can use information about a ligand (ligand-based) or its interaction with a desired target (structure-based) to predict other molecules that may have the same properties. To predict potential drug candidates, VS was performed using DrugBank (version 5.1.4) and FooDB (version pre-1.0) libraries. Various approaches have been used to obtain better results:

- Using LigandScout, ligand-based screening (using the pharmacophoric model of misoprostol) and structure-based screening (using interactions between misoprostol and EP3) were performed.
- Using AutoDock Vina, structure-based screening was performed using restricted docking (scoring different poses of the ligand in the chosen region and comparing the best scores of all molecules).

Next, a consensus was reached to harmonize all the approaches.

#### 4.2.1. LigandScout

The libraries used were converted using idbgen, a command available as part of LigandScout [50,51], and saved in the ldb file format. The targets were prepared using LigandScout’s GUI and saved in the pmz file format. For the ligand-based approach, the pharmacophoric model for misoprostol was generated after selecting the pose with minimal energy, and for the structure-based approach, the pharmacophoric model for the interaction of misoprostol with the EP3 receptor was used alongside the interaction details from the docking results. For each of these approaches, screenings were performed using MetaScreener, and the number of features to be automatically omitted to match the models (from 0 to 2). For each approach and number of allowed feature omissions, a table containing all compounds with their scores and ranks was automatically generated.

#### 4.2.2. AutoDock Vina

The libraries were prepared using AutoDockTools’ prepare_receptor4.py and saved in the pdbqt file format. The receptor was the same as that used in the blind docking step. Virtual Screening was performed on the previously identified region of the EP3 receptor for both libraries using AutoDock Vina through MetaScreener scripts, and the default parameters were employed to maximise efficiency. A table containing all compounds with their scores and ranks was automatically generated.

### 4.3. Consensus and Other Metrics

Combining data from different VS techniques can provide better overall results [52]. For this, the results of all screening techniques were joined into a single table and reordered, favouring compounds with higher ranks (top hits) on any screening and compounds with good scores among all screenings. Additional data, such as existing evidence in the literature about their targets (in order to skip already known compounds like prostaglandin-like compounds) or ADME/Tox properties (to promote drug-like compounds) (Table S3), were obtained for selected hits. After deliberation, selected compounds available in stock from different vendors for experimental validation were purchased.

The ADME/Tox properties were predicted using Maestro’s QikProp [53], with default settings.

### 4.4. Cell Culture and Drugs

The human endothelial cell line (EAhy926) was obtained from the American Type Culture Collection (ATCC; Rockville, MD, USA). The cell line was cultured in high-glucose Dulbecco’s Modified Eagle’s medium (DMEM) containing 10% heat-inactivated foetal bovine serum (FBS), 50 U/mL penicillin, and 50 µg/mL streptomycin (Sigma Aldrich Chemical Co., St. Louis, MO, USA) in an atmosphere of 5% CO_2_ and 95% humidified air at 37 °C. Subcultures were performed when 90% confluence was obtained.

Nordihydroguaiaretic acid (NOGA, Masoprocol), Hydrocortisone valerate, Pravastatin Sodium, Sodium Taurocholate (TUCA), and PGE_2_ were purchased from Molport (Letonia) and dissolved in dimethyl sulfoxide (DMSO; Sigma-Aldrich) up to 100 mM and stored at −20 °C.

### 4.5. Cell Viability Assay

Exponentially growing cells were plated in triplicate in flat-bottomed 96-well plates (Nunc, Roskilde, Denmark) at 2000 cells/well. The drugs were serially diluted to different concentrations. The control wells contained medium without the drug plus 0.1% DMSO. The plates were incubated for 3 days in a humidified 5% CO_2_ incubator and assayed for cell viability.

Thirty microlitres of 3-(4,5-dimethylthiazol-2-yl)-2,5-diphenyl tetrazolium bromide (MTT, Sigma-Aldrich, St. Louis, MO, USA) dissolved in phosphate-buffered saline (PBS) (final concentration 1.9 mg/mL, pH 7.4) was added to the cells. After incubation at 37 °C for 4 h, unreacted dye was aspirated. The formazan crystals were dissolved in 200 µL DMSO for 30 min, and the absorbance was read at 570 nm (test) and 690 nm (reference) in a microtiter plate reader (SpectraMax I3, Molecular Devices). The results were calculated as follows:

(1) $$cell\;viability\;\left(\%\right)=\frac{average\;O.D.of\;wells}{average\;O.D.of\;control\;wells}$$

All the experiments were run three times.

### 4.6. Cell Migration Assay (Wound Healing Assay)

Cell migration was studied using a wound-healing assay. Human endothelial cells (80,000 cells) were plated in low 35-mm dishes with culture inserts (Ibidi, Martinsried, Germany) in the presence of 10% FBS. After appropriate cell attachment for 24 h, inserts were removed with sterile forceps to create a wound field of approximately 500 µm according to the manufacturer’s protocol. Confluent cells were incubated with the following treatments: 20 µM NOGA, 0.75 mM TUCA, 5 µM Pravastatin sodium, 20 µM Hydrocortisone valerate, and 0.2 µM Iloprost. Control dishes were incubated with 0.1% DMSO and 20 nM PGE2. The cells were allowed to migrate in a cell culture incubator for 24 h. At 0, 4, 7, and 24 h (linear growth phase), 10 fields of the injured area were photographed with an inverted-phase contrast microscope at ×10 magnification. For each time point, the area covered by cells was determined by analysis using ImageJ software version 1.53 (National Institutes of Health, Bethesda, MD, USA). All experiments were repeated thrice.

The migration speed of wound closure is given as the percentage of the recovered area at each time point relative to the initially covered area (0 h). The rate of wound closure (%/h) was calculated using the formula:

(2) $$Slope\;\left(\frac{\%}{h}\right)=\frac{\left(\%\;covered\;area\;at\;t_{x}\right)-\left(\%\;covered\;area\;at\;t_{0}\right)}{t_{x}-t_{0}}$$

where slopes are expressed as percentages relative to the control conditions.

### 4.7. Ex Vivo Assessment of Platelet Activation and Platelet–Leukocyte Aggregates (PLAs)

Seven healthy participants (without any treatment) were recruited from the staff of the Catholic University of Murcia (UCAM) after obtaining approval from the UCAM Research Ethics Committee (CE02208). Fresh blood samples were collected in commercial 2 mL sodium citrate (32%) and EDTA Vacutainer tubes using a 20-gauge needle. Blood extractions were performed in accordance with the Declaration of Helsinki, and participants provided written informed consent.

Selected ligands were dissolved in PBS up to 1 mM and then added to 150 µL of fresh citrated blood at 20 nM PGE_2_, 10 µM NOGA, 0.75 mM TUCA, 10 µM pravastatin sodium, 10 µM hydrocortisone valerate, and 0.3 µM Iloprost for 10 min, followed by platelet activation marker analysis by flow cytometry (FCM). After incubation with EP receptor ligands for 10 min, whole blood (25 µL) was stimulated with adenosine-50-diphosphate (ADP, 0.02 mmol/L; Biodata Corporation, Horsham, PA, USA) for 2 min. Later, whole blood was labelled with mouse anti-human CD61-fluorescein isothiocyanate (FITC), mouse anti-human CD62P-BV786, and mouse anti-human CD42b-allophycocyanin (APC) (all from BD Biosciences, Oxford, UK) for 15 min, diluted with 1 mL of filtered PBS, and analysed using a FACS Celesta flow cytometer (BD, Becton Dickinson, Oxford, UK), as already published [12,54].

Selected ligands were then added to 50 µL of fresh EDTA-anticoagulated whole blood for 10 min. Mouse anti-human monoclonal fluorochrome-conjugated anti-CD14-PE and CD42b-BUV395 antibodies were mixed with 50 µL of stimulated whole blood in TruCount tubes (BD Biosciences). After incubation for 15 min, red blood cells were lysed with 450 µL of ammonium-chloride-potassium (ACK) lysing solution (BD, Oxford, UK) for 15 min, followed by dilution in 1 mL PBS and immediate flow cytometric analysis. Monocytes and other polymorphonuclear leukocytes (PMNs) were selected using gating strategies based on forward and side scatter to select monocytes and side scatter versus CD14 expression to exclude granulocytes (some lymphocytes and neutrophils might also be included in this gate) [12]. Absolute counts of CD14+ leukocytes (in cells per microlitre) were obtained by calculating the number of gated events proportional to the number of count beads, according to the manufacturer’s recommendations. CD14+ platelet–leukocyte aggregates (PLAs) were defined as events positive for CD14 and platelet marker CD42b [55]. Isotype controls for all FCM assays were performed using different monoclonal anti-IgG1 antibodies (BD Biosciences).

### 4.8. Data Analysis

Data are expressed as means ± standard deviation (SD). Data were analysed for statistical differences using Student’s *t*-test for paired and unpaired data after testing for normal distribution of the data. For in vitro/ex vivo experiments, one-way analysis of variance (ANOVA) was performed, followed by Tukey’s post hoc test to compare each group. Differences were considered significant at an error probability of *p* < 0.05. GraphPad software (version 10.0, GraphPad Software, San Diego, CA, USA) was used for all statistical analyses.

## 5. Conclusions

This study demonstrated the successful integration of computational and experimental strategies to identify novel EP3 receptor ligands with potential therapeutic applications in CVD. Ligand-based and structure-based virtual screening approaches, supported by the MetaScreener suite, have enabled the prioritisation of lead compounds with high docking scores and favourable predicted pharmacokinetic profiles. The appearance of known ligands among top results and the experimental validation confirmed the biological relevance of in silico predictions. Several compounds exhibit significant bioactivity in endothelial and platelet-based assays. Among these, TUCA, masoprocol, hydrocortisone, and pravastatin sodium have emerged as the most effective candidates, supporting the role of EP3 modulation as a viable cardiovascular strategy.

In particular, TUCA demonstrated the strongest anti-migratory activity, consistently outperforming other compounds in reducing endothelial cell migration. These results highlight its potential as a lead molecule, although further studies are required to elucidate its underlying mechanisms of action. In the context of platelet function, only TUCA platelet aggregation under basal conditions was observed, although reductions in CD61 and CD62P expression levels were not found in other conditions or ADP stimulation.

These findings support the hypothesis that EP3 ligands play a broader role in modulating vascular inflammation. The combined in silico and in vitro workflow not only streamlined candidate selection but also produced high-affinity ligands with minimal predicted off-target effects, reinforcing the need for subsequent pharmacodynamic and in vivo studies to advance these candidates for clinical translation.

Finally, this study highlights the dual potential of both drug repurposing and novel ligand development. Together, these findings open promising avenues for structure–activity relationship studies and rational design of next-generation EP3-targeted therapeutics.

## Figures and Tables

**Table 1:** Summary of top-ranked compounds identified through virtual screening. The AD column shows the binding scores (kcal/mol) obtained using AutoDock Vina. Columns labelled SB-{N} and LB-{N} represent LigandScout scores, where SB refers to structure-based pharmacophore models, and LB refers to ligand-based models. The number {N} following each label indicates the number of features allowed to be omitted during screening (0–2). Only the highest-scoring candidates from DrugBank and FooDB libraries are shown. Notable candidates used in the experimental validation, such as masoprocol, iloprost, and pravastatin, are highlighted.

Code	AD	SB-0	SB-1	SB-2	LB-1	LB-2	Name
FDB023044	−8.12	0.98	0.98	0.98	0.86	0.86	Prostaglandin F1a
FDB022853	−8.00	0.98	0.98	0.98	0.86	0.86	Prostaglandin F3a
FDB023346	−7.90	0.98	0.98	0.98	0.87	0.87	6-KetoPG ^1^ E1
FDB022647	−8.27	0.97	0.97	0.97		0.76	Prostaglandin F2b
FDB022851	−7.99	0.98	0.98	0.98		0.76	8-isoPG ^1^ F2b
DB00770	−7.76		0.89	0.97	0.87	0.87	Alprostadil
FDB022625	−7.71		0.89	0.97	0.87	0.87	Prostaglandin E1
DB12708	−9.34			0.89		0.78	Sulprostone
DB01240	−8.14	0.97	0.97	0.97		0.77	Epoprostenol
FDB022560	−8.06	0.97	0.97	0.97		0.77	Prostaglandin I2
DB11507	−9.22			0.81	0.87	0.87	Cloprostenol
DB00179	−8.76				0.86	0.86	**Masoprocol (NOGA)**
FDB022448	−8.11	0.98	0.98			0.76	Prostaglandin F2a
DB14544	−8.66				0.86	0.86	**HC ^2^ valerate**
DB01088	−7.99			0.97		0.76	**Iloprost**
DB06555	−13.08						Siramesine
FDB011293	−13.00						Sesamolinol
DB08512	−12.65						
DB07538	−12.55						
DB03583	−12.48						
DB00175	−8.25			0.81			**Pravastatin**
DB08964	−8.22				0.86	0.96	Gemeprost
DB00929	−8.19				0.87	0.87	Misoprostol
DB07528	−12.42						
DB06925	−12.36						
DB15345	−12.26						GSK-945237
DB07545	−12.17						
FDB000425	−12.15						
FDB023593	−7.36			0.81			Pravastatin
FDB023130	−8.22		0.90	0.90			Thromboxane B2
DB12043	−9.55				0.86	0.86	Sepetaprost
DB04348	−6.53			0.81			**Taurocholic acid (TUCA)**

^1^ PG = Prostaglandin, ^2^ HC = Hydrocortisone.

## Data Availability

The raw data supporting the conclusions of this article will be made available by the authors on request.

## Supplementary Materials

The following supporting information can be downloaded at: https://www.mdpi.com/article/10.3390/ijms26104879/s1.

## Abbreviations

The following abbreviations are used in this manuscript:

ADME/Tox	Absorption, Distribution, Metabolism, Excretion / Toxicity
GUI	Graphical User Interface
TUCA	Taurocholic Acid
CVD	Cardiovascular Disease
PGE_2_	Prostaglandin E_2_
NOGA	Nordihydroguaiaretic acid (Masoprocol)
MTT	Thiazolyl Blue Tetrazolium Bromide
PLA	Leukocyte–Platelet Aggregate
EDTA	Ethylenediaminetetraacetic Acid
PGI_2_	Prostaglandin I_2_
HMG-CoA	Hydroxymethylglutaryl-CoA
FBS	Fetal Bovine Serum
DMSO	Dimethyl Sulfoxide
PBS	Phosphate-Buffered Saline
PMN	Polymorphonuclear Leukocytes
