# Supplementary material for: Discovery and Functional Validation of EP3 Receptor Ligands with Therapeutic Potential in Cardiovascular Disease
Source: Int J Mol Sci. 2025 May 19;26(10):4879. doi: 10.3390/ijms26104879 (PMC12112076; doi:10.3390/ijms26104879)
Supplement: Supplementary file 1 [file ijms-26-04879-s001.zip › ijms-3609562-supplementary.pdf]

Supplemental Data

Table S1

Global Score	x	y	z
-8,27	129,92	-6,96	154,64
-8,24	129,73	-7,21	154,81
-8,24	129,74	-7,24	154,82
-8,23	129,70	-7,34	154,96
-8,23	129,72	-7,20	154,81
-8,23	129,74	-7,17	154,81
-8,23	129,67	-7,28	154,98
-8,23	129,85	-7,01	154,68
-8,22	129,72	-7,20	154,84
-8,22	129,64	-7,29	154,96
-8,22	129,74	-7,24	154,85
-8,22	129,63	-7,29	154,98

Table S1. Top-ranked binding poses of misoprostol at the EP3 receptor, obtained from blind docking (BD) calculations. All poses are located within the same region near the extracellular cavity, and are reported as part of the same cluster. The "Global Score" column represents the predicted binding energies (in kcal/mol) generated by AutoDock Vina. Columns "x", "y", and "z" indicate the cartesian coordinates of each pose. These results highlight a consistent high-affinity binding pocket for misoprostol at the extracellular interface.

Table S2

	55	58	103	106	107	110	114	133	137	140	144	206	207	209	234	295	298	329	332	333	335	336	339
name	PRO	MET	GLN	THR	THR	VAL	TYR	PHE	MET	PHE	SER	THR	TRP	PHE	PHE	TRP	LEU	LEU	VAL	ARG	ALA	SER	GLN
9-D1t-PhytoP	Hp	Hp	Hp+HB		Hp	Hp				Hp	HB	HB								SB			Hp+HB
**Taurocholic acid (TUCA)**		Hp	HB+Hp			Hp	Hp		HB	HB		HB	Hp					Hp		HB		HB	HB
Misoprostol			HB			Hp				Hp		HB	Hp					Hp	Hp	SB		HB	HB+Hp
**Prostaglandin E2**	Hp		Hp	HB	Hp						HB		Hp						Hp			HB	HB
**Hydrocortisone valerate**	Hp	Hp	Hp	Hp	Hp	Hp					HB			Hp	Hp		Hp		Hp	HB	Hp		HB+Hp
**Iloprost**						Hp	Hp	Hp		Hp			Hp			Hp		Hp		SB	Hp		
**Masoprocol (NOGA)**		Hp	HB+Hp			Hp						HB	Hp					Hp		HB			HB
Pravastatin	HB	Hp	Hp						HB				Hp					Hp	Hp	Hp	HB	HB	HB
L-798,106	Hp	Hp	Hp		Hp	Hp		Hp										Hp	Hp				HB

Table S2. Summary of interactions predicted by AutoDock Vina during BD or VS. Legend: Hp = hydrophobic; HB = hydrogen bond; SB = salt bridges.

Table S3

molecule	name	SASA	QP polrz	QP logPw	QP logS	QP logHERG	HOA	PHOA
DB04348	**Taurocholic acid (TUCA)**	783	47,2	19,2	-3,9	-1,4	2	38
DB06555	Siramesine	824	55,0	6,6	-8,6	-8,3	1	100
FDB000497	Betanin	804	43,0	35,8	-1,7	-4,5	1	0
DB00929	**Misoprostol**	819	42,4	8,9	-6,2	-5,5	1	95
DB02691	Glycocholic acid	766	46,1	18,6	-4,3	-1,4	2	50
DB14544	**Hydrocortisone valerate**	731	45,0	13,3	-4,9	-4,5	3	84
DB00175	Pravastatin	726	41	13,2	-4,3	-3,1	2	66
FDB023044	Prostaglandin F1a	762	36,9	12,5	-4,6	-3,5	1	68
DB01088	**Iloprost**	732	39,2	10,3	-5,5	-3,3	2	78
FDB011293	Sesamolinol	598	36,1	10,7	-4,0	-5,2	3	100
DB00179	**Masoprocol (NOGA)**	579	30,5	10,8	-3,6	-5,1	3	79

Table S3. Predicted ADME/Tox properties of selected candidate compounds. SASA indicates Solvent Accessible Surface Area (in Å²). QPpolrz is the predicted polarizability; QPlogPw and QPlogS represent predicted water-octanol partition coefficient and aqueous solubility, respectively. QPlogHERG reflects potential HERG channel inhibition (a cardiotoxicity marker), where more negative values indicate greater risk. HOA refers to qualitative Human Oral Absorption (scale: 1 = low, 2 = medium, 3 = high), and PHOA gives the corresponding Percent Human Oral Absorption. These in silico descriptors support early-stage drug-likeness evaluation. Notable candidates used in experimental validation such as masoprocol, iloprost, and pravastatin are highlighted.

Figure S1

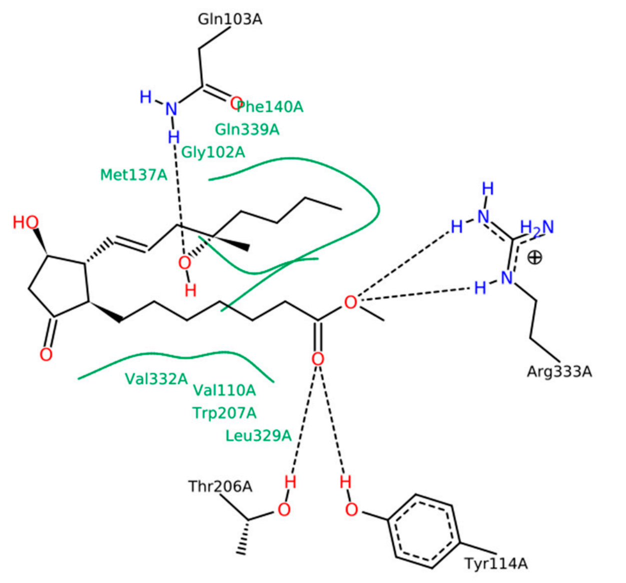

Figure S1. Sketch of the interactions generated by PLIP from the pose of misoprostol outputted by AutoDock Vina.

Figure S2

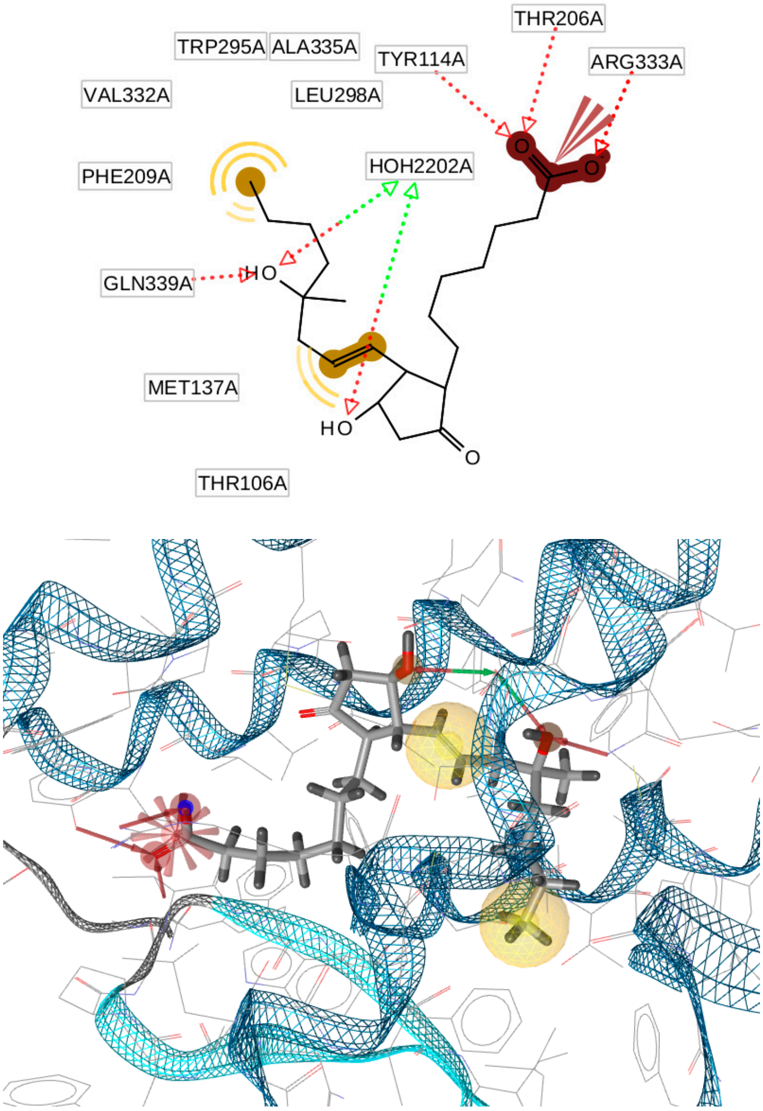

Figure S2. Pharmacophore made with interactions detected by LigandScout, used for SBVS, in 2D (left) and 3D (right) views. Legend: red dotted lines = H bond acceptors; green dotted lines = H bond donors; yellow highlight = hydrophobic; red highlight = negative ionizable.

Figure S3

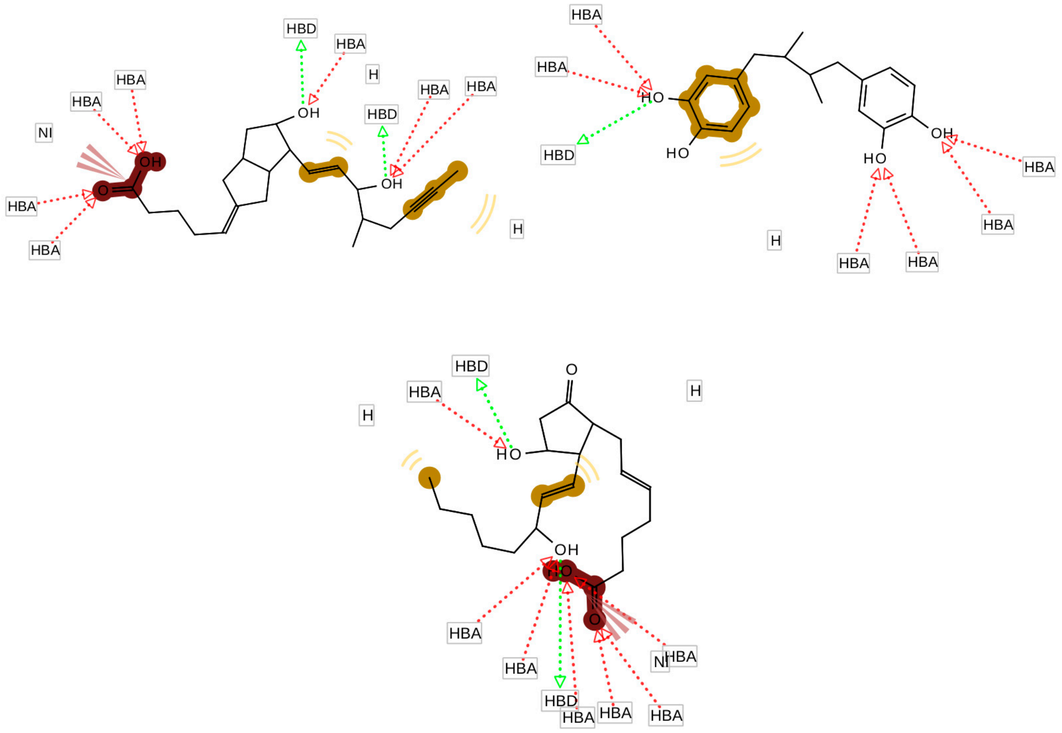

Figure S3. Pharmacophore overlayed on selected hits. From left to right: iloprost, NOGA, PGE2. Same legend as figure S1.
